# Evolution of echovirus 11 in a chronically infected immunodeficient patient

**DOI:** 10.1371/journal.ppat.1006943

**Published:** 2018-03-19

**Authors:** Majid Laassri, Tatiana Zagorodnyaya, Sharon Hassin-Baer, Rachel Handsher, Danit Sofer, Merav Weil, Konstantinos Karagiannis, Vahan Simonyan, Konstantin Chumakov, Lester Shulman

**Affiliations:** 1 FDA Center for Biologics Evaluation and Research, Silver Spring, MD, United States of America; 2 Movement Disorders Institute and Department of Neurology, Sheba Medical Center, Tel Hashomer; and Sackler Faculty of Medicine, Tel-Aviv University, Tel-Aviv, Israel; 3 Central Virology Laboratory, Public Health Service Laboratories Israel Ministry of Health at Sheba Medical Center, Tel Hashomer, Israel; 4 Department of Biochemistry and Molecular Biology, George Washington University Medical Center, Washington, DC, United States of America; 5 Dept. of Epidemiology and Preventive Medicine, School of Public Health, Sackler Faculty of Medicine, Tel Aviv University, Tel Aviv, Israel; National Institute for Biological Standards and Control, UNITED KINGDOM

## Abstract

Deep sequencing was used to determine complete nucleotide sequences of echovirus 11 (EV11) strains isolated from a chronically infected patient with CVID as well as from cases of acute enterovirus infection. Phylogenetic analysis showed that EV11 strains that circulated in Israel in 1980-90s could be divided into four clades. EV11 strains isolated from a chronically infected individual belonged to one of the four clades and over a period of 4 years accumulated mutations at a relatively constant rate. Extrapolation of mutations accumulation curve into the past suggested that the individual was infected with circulating EV11 in the first half of 1990s. Genomic regions coding for individual viral proteins did not appear to be under strong selective pressure except for protease 3C that was remarkably conserved. This may suggest its important role in maintaining persistent infection.

## Author summary

We describe evolution of Echovirus 11 genome in chronically infected immunodeficient patient over a period of several years and compare it with the evolution of circulating echoviruses from which it originated. Ratio of silent to missense mutations in protein coding regions suggests that chronic virus was under lower selective pressure than circulating viruses, except for a region coding for viral protease that may participate in neutralizing host cell anti-viral defense mechanisms. This suggests that adaptation to persistence in immunodeficient host may require maintaining functional viral counter-defense mechanisms.

## Introduction

Enteroviruses widely circulate in human populations and only rarely cause clinical disease. Polioviruses were the first enteroviruses to be isolated and the first to have the pattern of changes in their genome during chains of transmission characterized [1, 2]. They cause acute flaccid paralysis of limbs and occasionally bulbar paralysis. Paralysis rate in naïve individuals, however, is around one clinical case per 100 to 1000 infections. Other enteroviruses can also cause paralysis but at a lower rate. Enteroviruses cause a variety of other clinical conditions including mild fever, hand-foot-and-mouth disease, herpangina, myocarditis, diabetes, etc.

Human echovirus 11 is a member of the human enterovirus B species and is one of the most commonly isolated enteroviruses [3, 4]. EV11 viral capsid protein 1 (VP1) sequences segregate into six or more genogroups [5, 6]. Data from the Annual Reports of the Central Virology Laboratories, Public Health Service of the Israeli Ministry of Health, Tel Hashomer Israel, indicate that EV11 is endemic in Israel with at least one EV11-positive case being reported on 17 of the 27 years between 1986 and 2012. There was a major peak of 90 EV11-positive stools in 1999 and smaller peaks of in 1991, 1993, 1995–6, 2001, 2005 and 2011.

Enteroviruses are also able to chronically infect individuals with several kinds of primary immunodeficiency [7–10], and persist for months or years being regularly excreted in stool. This phenomenon is best known for poliovirus, which belongs to species C of human enteroviruses. Chronic poliovirus infection can last for over 30 years [11]. At least 110 immune deficient individuals have been identified who had persistent infections with immunodeficiency-associated vaccine-derived polioviruses (iVDPVs) [12, 13]. Additional cases of persistent infection from unidentified individuals have been inferred from environmental surveillance and isolation of ambiguous highly evolved vaccine-derived polioviruses (aVDPVs) [14]. Circulating vaccine-derived polioviruses (cVDPV) represent another kind of VDPV [15]. They have regained virulence and the ability to transmit in human populations indistinguishable from wild polioviruses, and can serve as a model for natural evolution of enteroviruses. VDPVs can cause outbreaks of paralytic disease in susceptible immune competent individuals. The pattern of amino acid substitutions and recombination of iVDPVs and aVDPVs has been shown to differ from cVDPV [16].

Among the EV11 isolates from Israel were 10 referred to hereafter as iEV11s that were obtained from the CSF of an immune deficient individual with Common Variable Immunodeficiency (CVID) who suffered from symptoms of chronic encephalomyelitis between 1995 and 1999. VP1 sequences of these isolates were obtained using Sanger-based sequencing. These sequences were compared with VP1 sequences EV11s isolated from cerebrospinal fluid, stool, rectal and throat swabs of contemporary cases of EV11 infections of non-immune deficient patients (2 in1992, 3 in1993, 1 each in 1996, 1997 and 1998, and 8 in 1999). A comparison of the patterns of differences in the VP1s of the two groups indicated that the iEV11s represented a persistent infection rather than repeated re-infections from contemporary EV11s circulating in the community.

Here we report the use of deep sequencing to determine complete genomic sequences of the 10 iEV11 isolates and 16 contemporary cEV11s to confirm that the iEV11s represented a persistent infection and to obtain information on the pattern of its evolution in humans during persistent infections of immune deficient individuals. To our knowledge this is the first analysis of evolution of HEV B during a persistent infection using complete genome sequences.

## Materials and methods

### Ethics statement

The Ethical Review Board of the Sheba Medical Center, Tel Hashomer approved this study (SMC-7685). The clinical samples from which viruses were isolated are a part of the virus strain collection of the Central Virology Laboratory in Israel. They were received between 1986 and 2014 and results were stripped of all links to personal details pertaining to, or which could be used to identify individual patients. All data were analyzed anonymously. The Ethical Review Board exempted this study from a requirement for obtaining informed consent.

### Sources of viruses

The annual incidence of laboratory confirmed EV11-positive stools received by the Central Virology Laboratory in Israel between 1986 and 2014 is presented in S1 Fig. Altogether there were 212 individuals with EV11-positive clinical samples. Among them were eleven EV11 isolates obtained between November 13, 1995 and December 21, 1999 from a male patient. These isolates are referred to as iEV11s to indicate that they were isolated form an immune deficient individual. The patient, born in 1955, was diagnosed with CVID at age 20 after recurrent bouts of pneumonia, sinusitis, and ear infections along with episodes of aseptic arthritis, as well as leukopenia and alopecia areata advancing to alopecia universalis. His younger brother had died from a bacterial complication known to be associated with CVID. The patient was treated with intravenous immunoglobulins (IViG) between 1975 and 1995, and prophylactically with antibiotics between 1985 and 1995. Following a year or more of poor adherence to IViG treatment, decreasing the infusions to once in 2 months, he presented with decreased hearing, a rash and neurological symptoms of headaches, stroke-like episodes and proximal lower limb weakness. He was diagnosed at the department of Neurology as suffering from chronic enteroviral meningoencephalomyelitis due to EV-11 that did not respond to reintroduction of frequent (bi-monthly) IViG infusions. In 1998 the patient received two one-week courses of daily Pleconaril (400 mg/day), under expanded access in February and then again in October. This treatment did not affect the course of his disease and his lower limb weakness gradually deteriorated along with mild cognitive difficulties. In December 2012 there was sudden worsening of his condition and he lapsed into a coma associated with acute hydrocephalus that did not respond to a ventriculoperitoneal shunt and he succumbed to his illness.Representative EV11 isolates collected prior to and during the isolation of these iEV11 samples that were sent for clinical analysis were chosen for comparison: eight from sporadic cases presenting between 1992 and 1998 and eight collected during a large EV11 outbreak in 1999. Clinical samples and symptoms associated with each isolate are indicated in S1 Table. Virus from the first tissue culture passage (TC1) that had been kept at -20°C were passaged once (TC2) on human human kidney epithelial cells (HuKi) received from Dr. Furin (Department of Health and Welfare, Ottawa, Canada) and RD cells (human rhabdomyosarcoma cell line; ATCC CCL136). Virus from four samples from the CVID patient and seven from among the representative samples were no longer viable by the time of this analysis (“Original TC” in S1 Table).

### Nucleic acid extraction

RNA was extracted from the virus in 1 ml of supernatant from the second passage for viable EV11 isolates, or from 1 ml of the original frozen stock for non-viable EV11 using a MagNA Pure LC2.0 Automatic extractor with MagNA Pure LC Total Nucleic Acid Isolation kit-High Performance (Roche Diagnostics, IN, USA) and eluted into 50 µl of elution buffer according to manufacturer’s instructions.

### Full length genomic sequences

The RNA library was prepared from the total RNA of 27 EV 11 isolates, the NEBNext mRNA Sample Prep Master Mix Set 1 (New England BioLabs, Ipswich, MA) was used according to the manufacturer's protocol (NEB). Briefly, 0.5 µg of the total RNA was used for fragmentation by focused ultrasonicator (Covaris) to generate the fragments of optimal sizes (250–300 nt) suitable for Illumina sequencing. cDNA was synthesized using SuperScript III Reverse Transcriptase (Invitrogen) and random primers. The cDNA was converted into double stranded cDNA followed by end repair procedure (Klenow fragment, T4 polynucleotide kinase and T4 polymerase), and was ligated to Illumina paired end (PE) adaptors.

Size selection was performed using double AMPure bead selection step (Beckman Coulter), generating DNA libraries ranging from 200 to 500 bp in size. Next the DNA libraries were expanded using 15 cycles of PCR with multiplex indexed primers and purified by magnetic beads (Agencourt AMPure PCR purification system, BeckmanCoulter). Finally, the DNA fragments were analyzed for quality and size distribution by BioAnalyzer using a high sensitivity kit (Agilent Technologies, Inc., Santa Clara, CA).

Deep sequencing was performed using MiSeq (Illumina) producing paired end reads of 250 nt long, or HiSeq2000 (Illumina) producing 101 nt long paired-end reads. The sequencing reads were analyzed by in-house 'swarm' or High Performance Integrated Virtual Environment (HIVE) software [17, 18]. Raw sequence reads were subjected to quality control and reads with phred score below 30 were removed. The reads were aligned to a curated database of 500 reference enteroviruses. Next, aligned sequences were separated into discrete sub-populations [19] and assembled into full length EV11 consensus genomic sequences (see S1 Table). This resulted in complete or near-complete genomic sequences of 25 isolates. For some virus isolates the depth of coverage (S1 Table) at the ends of genome was insufficient for reliable sequence reconstruction. To assemble individual consensuses of viruses present in mixtures a new algorithm was used [19]. The names of virus isolates were shortened to the name of EV11 genogroup followed by an incremental number by incremental order in which they were isolated.

### Molecular analyses

Neighbor-joining phylogenetic comparisons were prepared by Clustal X in MEGA v5.22 [20] or MacVector software using the Tamura-Nei substitution model [21]. Separate analyses were prepared for near-complete sequences with 5'-ends trimmed to the longest sequence common to all sequences in the alignment, the entire open reading frame, the P1 region encoding all four viral capsid proteins, Viral Capsid protein 1 (VP1), the P2 plus P3 coding region encoding all non structural proteins, the 3D RNA-primed RNA polymerase, and the 3’ untranslated region (3’UTR, also partly truncated). The initial alignments of these regions were prepared for MEGA using the Sequencher v5.2.2 program (Gencodes, Anne Arbor, MI, USAS).

Three-dimensional folding models of Loop V of the internal ribosomal entry site (IRES) in the 5’UTR were prepared using the web-based Unified Nucleic Acid Folding and hybridizing Package [22, 23] at the University of Albany NY, USA, (http://mfold.rna.albany.edu; last accessed November 2014) and the consensus sequence for the Israeli isolates. The three-dimensional folding model for the human enterovirus Z domain in the 3’UTR was taken from Merkel et. al. [24].

Amino acid differences between Israeli EV11 isolates from EV11 genogroup A and the earliest isolate from this genogroup, EV11-A01, were determined using the Sequencher program. Amino acid differences that became fixed during the persistent EV11 infection in the CVID patient were mapped onto the 5 capsomers located at the 5-fold axis of symmetry on the X-ray diffraction crystalline structure of the EV11 capsid (Accession number 1H8T) using the MacPyMol program (The PyMOL Molecular Graphics System, v7.2.1, Schrödinger, LLC., [25].

### Accession numbers

Genomic sequences were deposited in the GenBank/EMBL/DDBJ database (accession numbers KY981557 to KY981581), and the raw Illumina data in sequence read archive (https://hive.biochemistry.gwu.edu/review/Echovirus11).

## Results

### Determination of genomic sequences using direct RNA library deep sequencing approach

To determine genomic sequences of EV11 isolates (S1 Table) we have used direct RNA library deep sequencing approach because the amount of live virus in some samples was very low, and the quantity of isolated RNA would not be sufficient for multiple PCR reactions needed for conventional Sanger sequencing. To recover RNA from these samples carrier tRNA was added. RNA was then fragmented by focused ultrasonication, converted to double-stranded cDNA and sequenced using Illumina Miseq or Hiseq technology. This resulted in a variable level of sequencing coverage in most cases ranging from 1,000 to 250,000 with the exception of four samples in which the coverage was lower (See S1 Table). However, even for the sample with the lowest depth of coverage (40) we were able to assemble consensus sequence that had an uninterrupted open reading frame of 2,195 amino acids. The nature of RNA library preparation is such that there is a gradual decline of the number of molecules representing the very ends of the genome. In some cases the low coverage of the terminal regions resulted in consensus sequences that missed few nucleotides from the ends because they could not be reconstructed reliably.

The use of this protocol proved to be remarkably effective and the level of sequencing coverage per each nucleotide was sufficient not only for unambiguous reconstruction of consensus sequences but also for determining sequence heterogeneity profiles.

### Phylogenetic analysis

**Fig 1:**
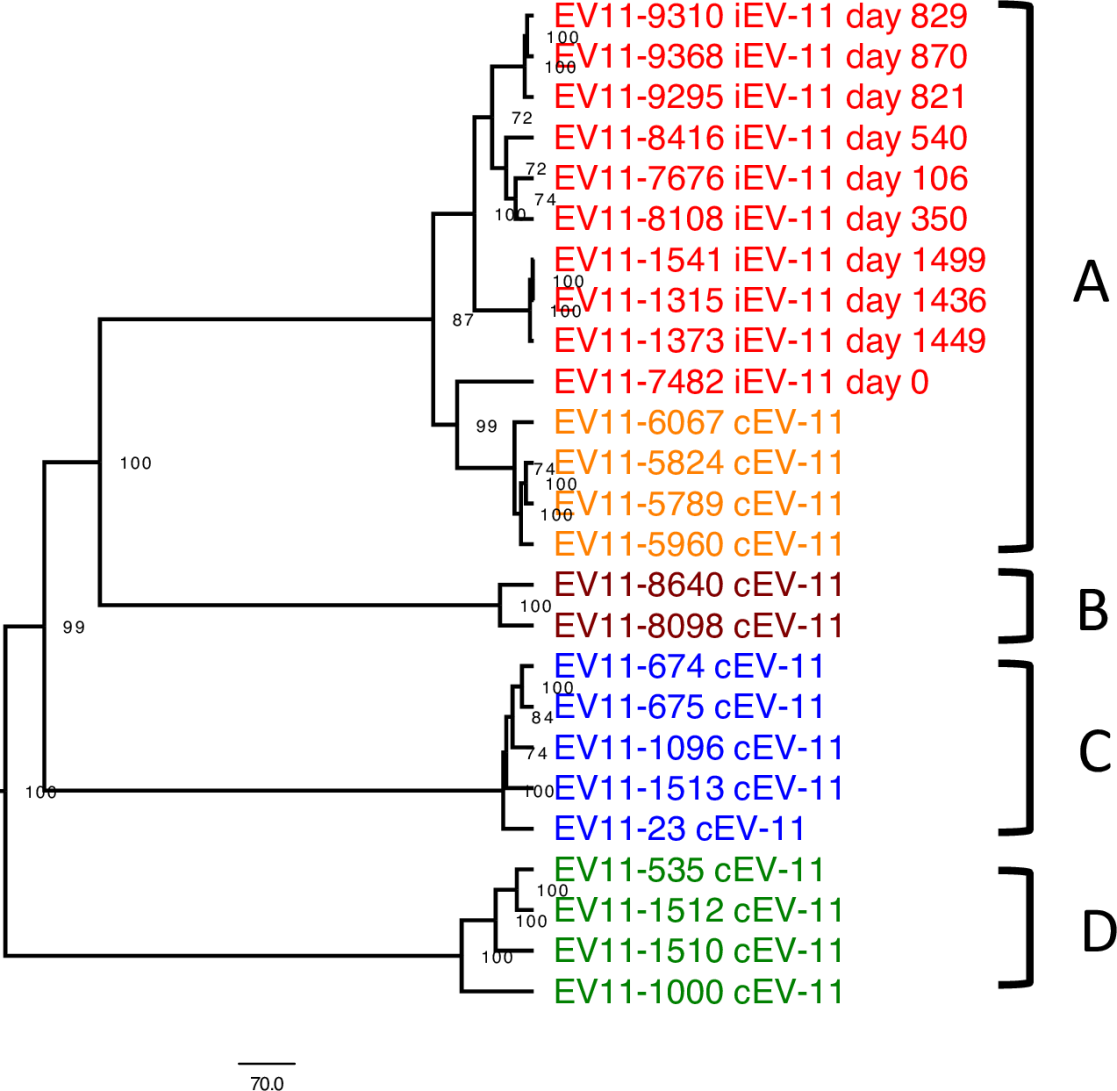
Phylogenetic relationship between full-length sequences of EV11 strains isolated in Israel in 1992–1999. iEV11 strains belonging to genogroup A are shown in red, cEV11 of genogroup A are shown in orange, while genogroups B, C, D in purple, blue, and green, respectively. The tree was constructed using unweighted pair group method with arithmetic mean (UPGMA). Bootstrap values (N = 1000) are indicated near the internal nodes of the tree.

**Fig 2:**
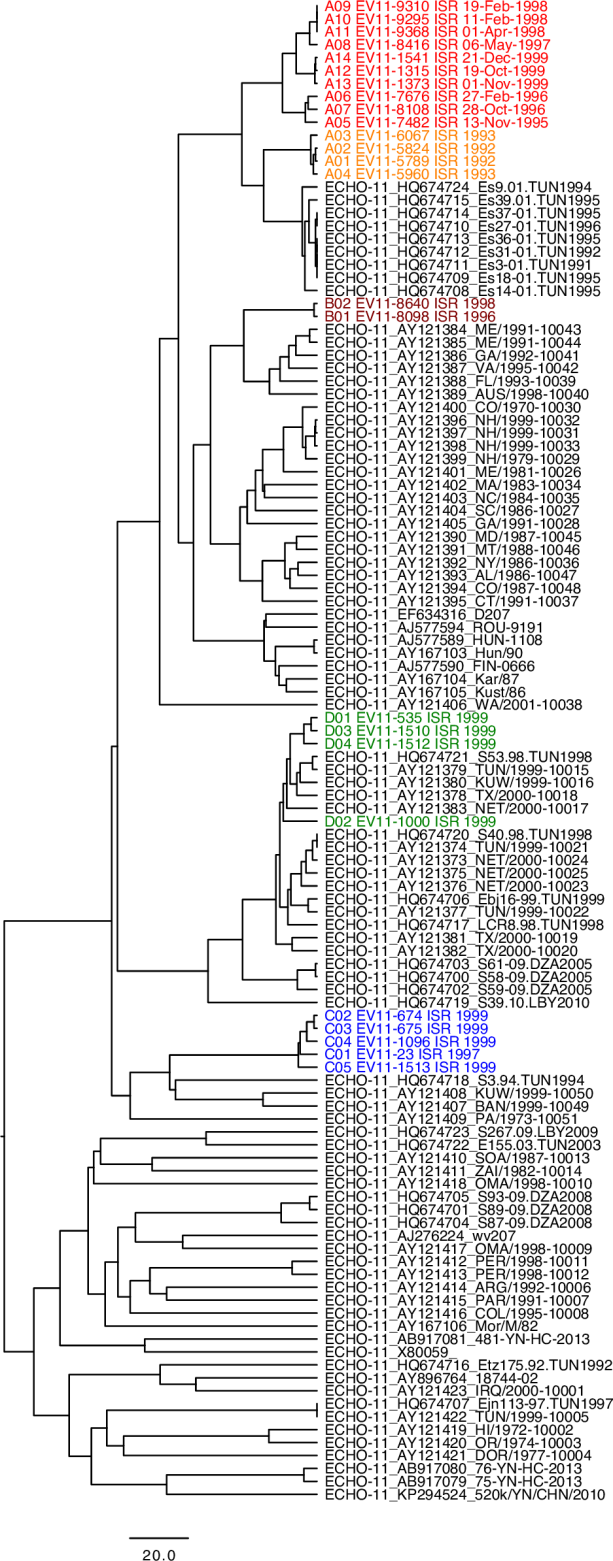
Phylogenetic tree based on nucleotide sequences of VP1 capsid protein showing the relationship between sequences determined in this study and all published EV11 sequences. Color codes are the same as in Fig 1. The tree was constructed using UPGMA algorithm.

The comparison of complete or near-complete genomes the EV11 isolates revealed that they could be separated into four clades or genogroups labeled A through D (Fig 1). Their relationship with other EV11 was determined by comparing with a representative subset of 100 published sequences of virion protein VP1 (Fig 2). On this and subsequent dendrograms the incremental number after the genogroup indicates the temporal order in which they were isolated. Attempts were made previously to classify EV11 isolates into genogroups [5, 26]. The comparison of the newly sequenced isolates showed that the closest relative to our genogroup A was 1995 Tunisian isolate (HQ674714), which had 3.4–3.7% nucleotide differences with cEV11, and 5.8–8.3% with iEV11. This means that our genogroup A matches genogroup IV-4 of Fares. Genogroup B was related to 1991 isolate from Maine (AY121384) with 4.3–4.6% difference, placing it into genogroup IV-4 of Fares and D4 of Oberste. Genogroup C was related at 11.2% to 1994 Tunisian isolate HQ674718 and at 11.1–11.5% to 1999 isolate from Kuwait (AY121408), belonging to Fares genogroup IV-3 and Oberste genogroup D3. Finally our genogroup D was very close (1.1–1.8%) to 1998 Tunisian isolate HQ674721 (Fares genogroup IV-5, Oberste genogroup A).

**Fig 3:**
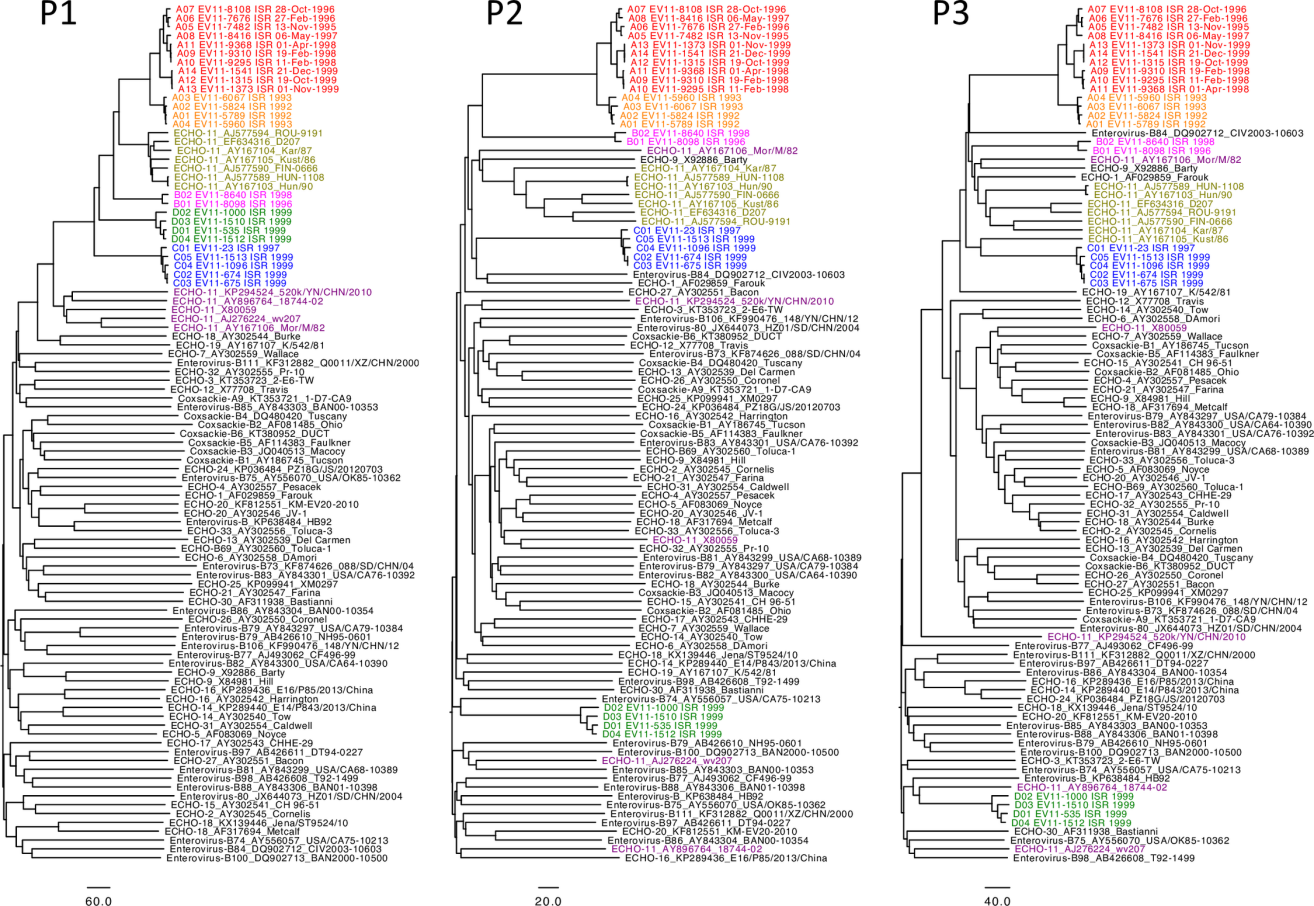
Phylogenetic trees constructed based on P1, P2, and P3 regions of EV11 genomes determined in this study along with reference HEV-B sequences. The trees were constructed using neighbor-joining method of Saitou and Nei [46].

Enteroviruses frequently recombine with other viruses of the same species. To determine whether the Israeli isolates are also products of recent recombination we have compared them with a reference set of 72 full-length sequences of Species B Enterovirus that contained at least one representative from each serotype, and all 12 full-length genomic sequences of EV11 available in Genbank. Figs 3A, 3B and 3C show the phylogeny of P1, P2, and P3 regions providing no evidence of recombination within the group of isolates from immunodeficient patient. In contrast, analysis of circulating strains revealed potential recombination event between P1 and P2 regions of genogroup D, as evidenced by its “jump” to another branch of the phylogenetic tree.

**Fig 4:**
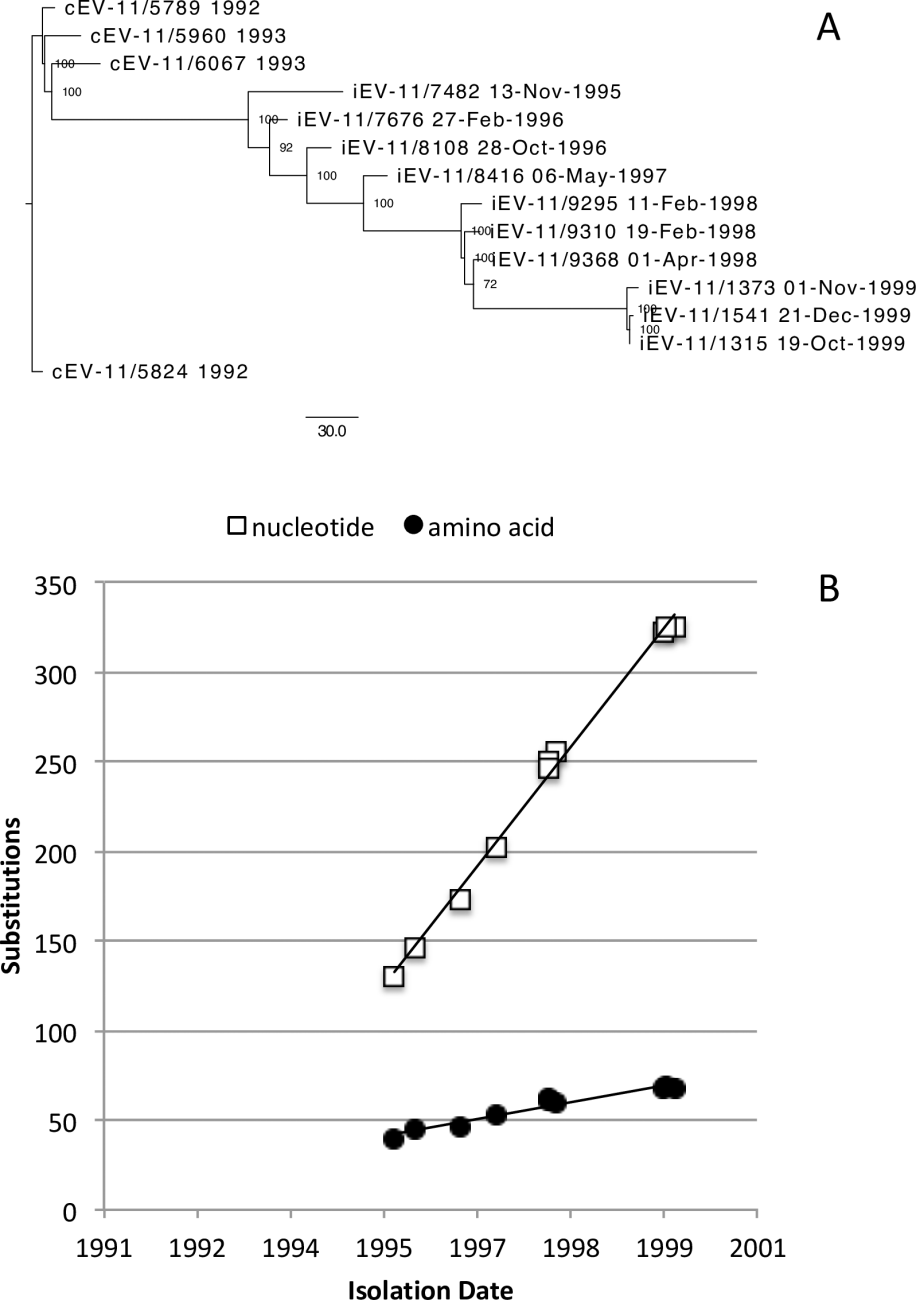
Gradual accumulation of mutations in iEV11 illustrated by neighbor-joining tree (A) and plots of the number of nucleotide and amino acid substitutions vs. time of isolation (B). The analysis was performed for the entire genome of these strains relative to the closest cEV11 isolate. Phylogenetic tree was constructed with neighbor-joining method and bootstrap values (N = 1000) are shown near the internal nodes of the tree.

Previous studies found about 30% divergence between different isolates of EV11 [26]. Therefore close similarity between strains described in this study lends support to the notion that all the iEV11s isolated between 1995 and 1999 were sequentially related to four group A isolates from 1992 and 1993. The hypothesis that these 1992 and 1993 isolates were closely related to the iEV11s is strengthened by the observation that cEV11 of genogroup A were closest to the earliest iEV11 isolate (5.8%), and more distant (8.3%) from the latest ones. This is illustrated by neighbor-joining phylogenetic tree shown on Fig 4A. It suggests that iEV11 may have evolved from a cEV11 strain close to the Israeli isolates from 1992–1993. Regression lines for the accumulation of nucleotide and amino acid substitutions relative to the closest cEV111 strain shown on Fig 4B can be extrapolated to the early 1990s, a tentative time when the patient became chronically infected with EV11.

The first iEV11 strain isolated in 1995 (A5) had a total of 139 nucleotide differences from the closest 1992 cEV11 isolate A1. These mutations never reverted back, and remained in all subsequent iEV11 isolates. An additional 12 “substitutions” that emerged during the same period, reverted back in subsequent iEV11 isolates. Between February 1996 and April 1998 there were 113 additional mutations that emerged and became fixed in all subsequent isolates and 8 more that emerged but then “reverted back” by October 1999. An additional 79 substitutions emerged between April 1998 and October 1999 followed by 2 more between October and December of 1999. Therefore during the four time intervals between 1995, 1996, 1998, and two dates in 1999 the rates of mutations fixation were: 0.83*10^−2^, 1.16*10^−2^, 0.71*10^−2^, and 0.68*10^−2^ mutations / site / year, a rate close to the molecular clock rate of 1.03*10^−2^ reported for circulating poliovirus [1], 1.51*10^−2^ for iVDPV [11], and 0.84*10^−2^ for EV30 isolated from chronically infected immunodeficient patient [8].

### Accumulation of amino acid differences

Analysis of the predicted amino acid substitutions that occurred during the iEV11 evolution showed that most (28 of 35, or 80%) of the amino acid substitutions became fixed in the evolving genome (S2 Table). Fixation of 10 amino acid substitutions resulted from 2 nucleotide differences in the codon, one involved 3 nucleotide differences, and one fixed amino acid was represented by a single nucleotide difference followed by a second silent mutation in the six subsequent isolates. Four amino acid differences were unique, e.g., found in only one of the 10 isolates, and one substitution appeared in isolates A06 through A10, but not A11 through A14.

**Fig 5:**
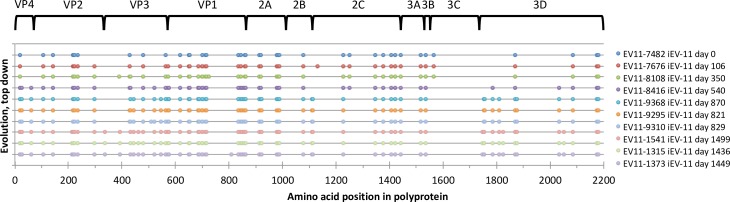
Distribution of mutations in the entire open reading frame of iEV11. Mutations were identified by comparing with the closest contemporary cEV11 isolate A01-cEV11-5789. The top line represents mutations that may have occurred prior to the infection of the patient, and all subsequent lines illustrate virus evolution in the patient.

The distribution of amino acid changes along the iEV11 genome is shown on Fig 5. It revealed that the entire 3C protein was highly conserved, along with parts of 3D (between amino acids 142 and 300), 3A (amino acids 1–128 and 140–236), 2C (amino acids 2–118), and VP1 (amino acids 157–267). Hot spots of mutations included amino acids 116–144 and 267–292 of VP1, and 145–164 of VP2.

**Fig 6:**
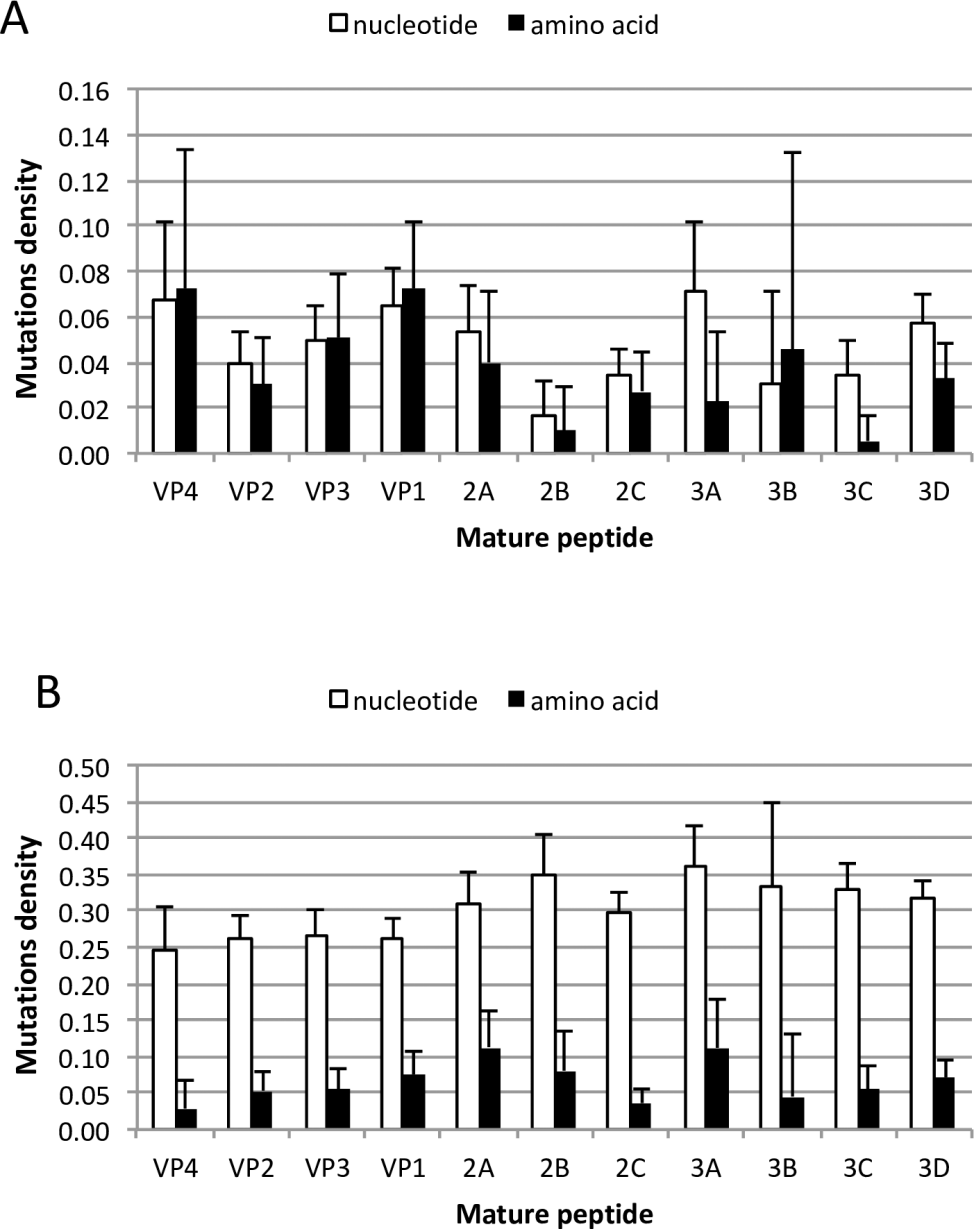
Amino acid and nucleotide substitutions densities (Da and Dn respectively) for individual parts of EV11 genome for iEV11 (A) and cEV11 (B). Error bars show the 95% confidence limits for binomial proportions.

The analysis of selective nature of mutations in individual viral proteins was performed by calculating density of nucleotide and amino acid substitutions, Dn and Da, respectively. The values represent the number of nucleotide and amino acid substitutions that have been fixed in at least one isolate, divided by the size of the coding region (nt) or the protein (aa). If the ratio Da/Dn is less than 1, then there is a negative selection against amino acid changes, while the ratio greater than 1 indicates possible positive selection of certain amino acid changes. Fig 6A shows that amino acid substitutions in most viral proteins were largely neutral, except for proteins 3C (protease) and 3D (RNA polymerase) (p-values 0.0366 and 0.0332), and marginally significant for 3A (p-value of 0.0910) that were under negative selective pressure. Amino acid sequence conservation was the strongest for 3C protease, in which only 1 out of 183 amino acids reverted from threonine at position 15 to alanine present in cEV11 strain A01-EV11-5789. In contrast, Dn and Da values for all genes of circulating EV11 differed significantly (p<0.001), suggesting that the selective pressure at amino acid level was higher and the conservation of proteins was more uniform across the genome (Fig 6B).

**Fig 7:**
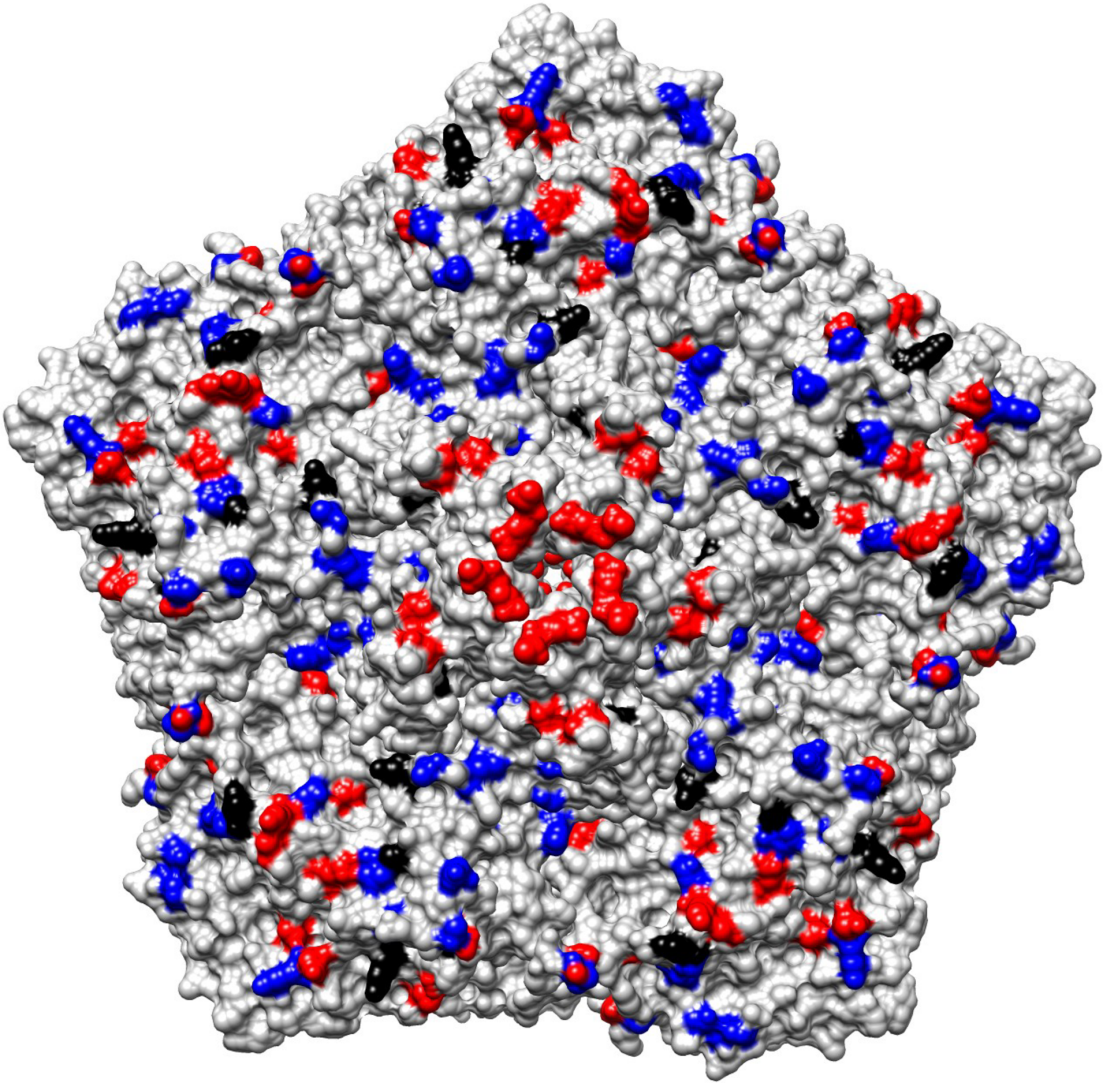
Amino acid differences mapped on the virion pentamer outer surface. Differences in iEV11 are shown in red, cEV11 in blue, and those that occurred in both are shown in black. Differences determined relative to isolate A01-EV11-5789 were mapped onto the three dimensional crystallographic model of the EV11 capsid (PDB ID: 1H8T).

The predicted amino acid difference among iEV11s relative to the earliest cEV11 isolate A01 from 1992 were mapped onto the three dimensional crystallographic model of the EV11 capsid (Protein Data Bank, PDB ID 1H8T). The fixed amino acid substitutions are shown for the capsid pentamer centered around the five-fold axis of symmetry (Fig 7). Many of the substitutions were on the outer capsid surface and the walls of the canyon, a circular depression surrounding the 5-fold axis of symmetry and important for high affinity binding to host cell receptors [27]. When only iEV11s were compared, most amino acid substitutions accumulated on the northern rim of the canyon next to the 5-fold axis of symmetry. There were 7 amino acids differences in P1 between A01-cEV11-5824 isolated in 1992 and the earliest iEV11, A05-iEV11-7482, isolated in 1995. The same was true for another cEV11 isolate from 1992 A02-cEV11-5789. The amino acids that differed were: N18S, R106C, D143G, E217D, T220A, A223V and H697C. (Notation: amino acid in cEV11 from 1992, the number of the amino acid position in P1, amino acid in iEV11 from 1995.)

### Nucleotides differences in domain V of the IRES in the 5’UTR

**Fig 8:**
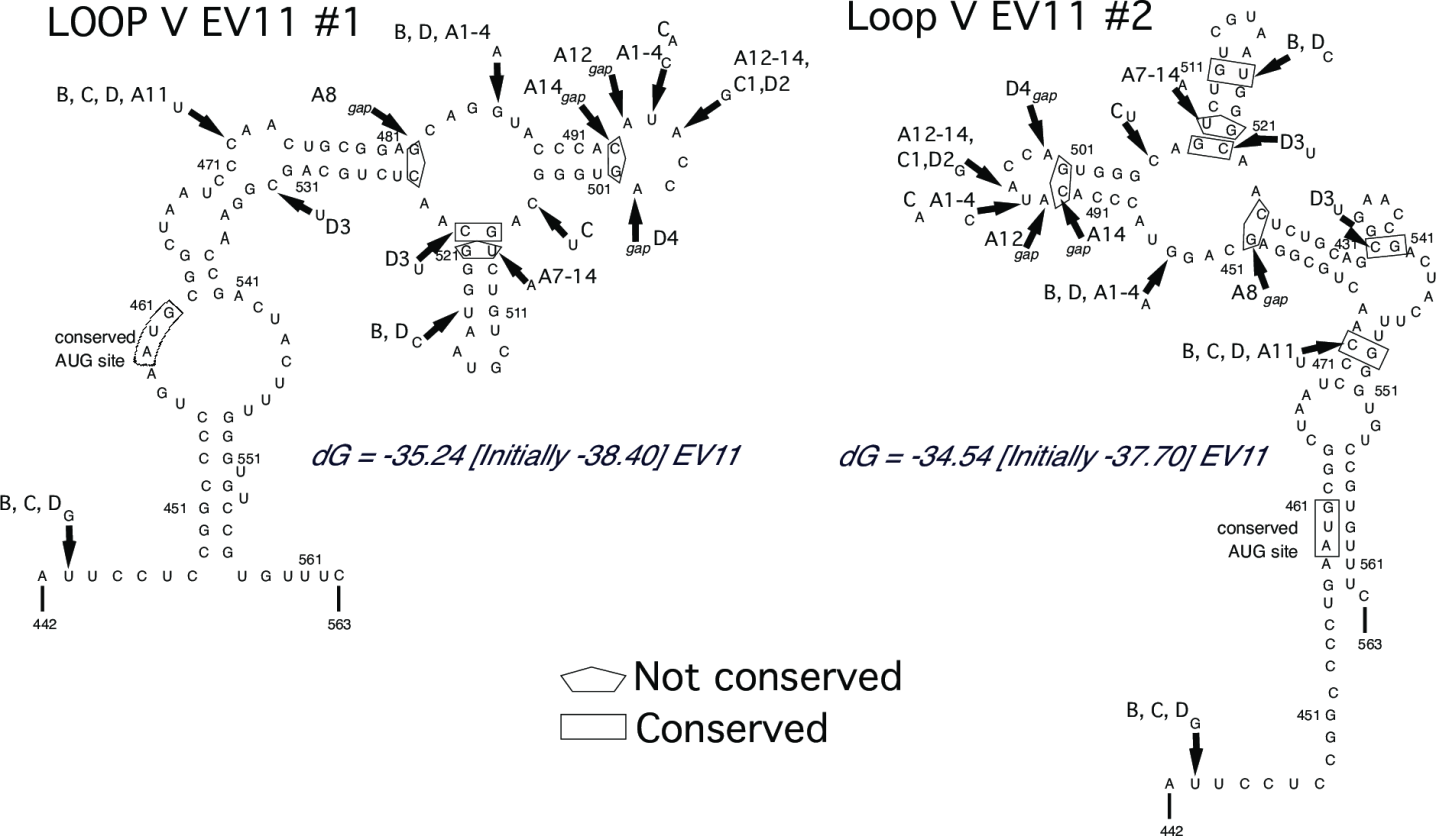
Predicted secondary structure of domain V of EV11 IRES element. Two best models are presented on panels A and B. Three-dimensional folding models of Loop V of the internal ribosomal entry site (IRES) in the 5’UTR were prepared using the web-based Unified Nucleic Acid Folding and hybridizing Package [17, 18] at the University of Albany NY, USA, (http://mfold.rna.albany.edu; last accessed November 2014) and the consensus sequence for the Israeli isolates.

The 5’-untranslated region of Enterovirus genome contains the internal ribosome entry site (IRES) [28, 29]. Analysis of iEV11 sequences revealed that between 1998 and 1999 a 13–14 nucleotide deletion became fixed in the hypervariable region of the 5’-UTR. Domain V in the IRES has been shown to be critical for enterovirus replication and virulence [30]. Stem and loop structures were predicted for RNA sequences of the all Israeli EV11s equivalent to domain V of poliovirus using the m-Fold program. Two highest probability stem-loop structures are shown in Fig 8. Arrows indicate each position where one or more of the Israeli isolates had an alterative nucleotide, and a pentagram or rectangle indicates whether the difference would disrupt or conserve the stem structure, respectively. In the first model one pair was conserved and three disrupted, while in the second model, four pairs were conserved and three disrupted. The overall shape of domain V predicted by the second model is similar to the shape of domain V of polioviruses.

### Nucleotide differences in Z domain of the 3’UTR

**Fig 9:**
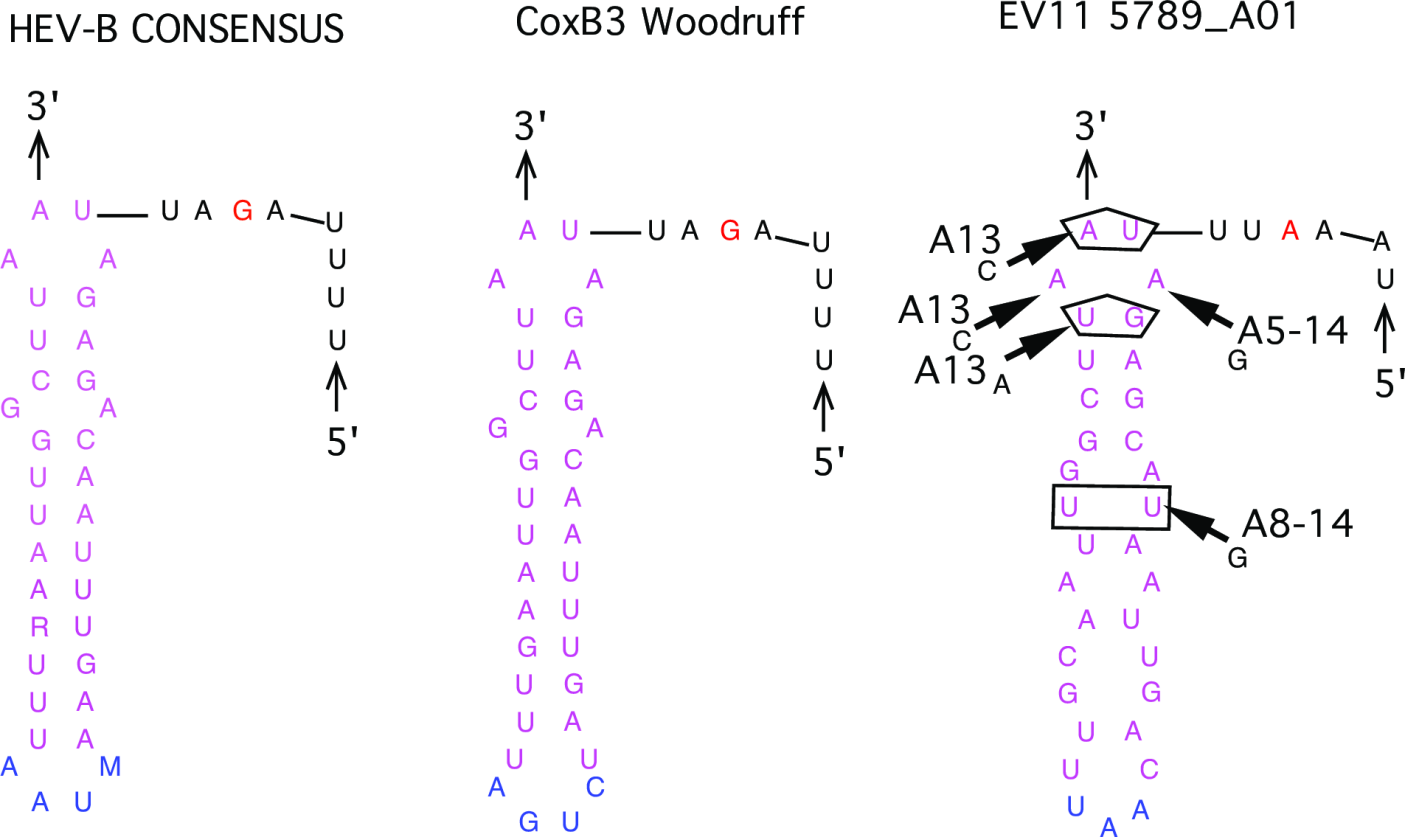
Predicted secondary structures of Z-domain in the 3'-UTR for HEV-B and Coxsackie B3 [24] and iEV11-5789 determined in this paper. The Israeli sequences were superimposed on the three-dimensional folding model for the human enterovirus Z domain in the 3’UTR taken from Merkel et. al. [24].

A cloverleaf structure at the 5’ end of the 3’-UTR serves as the specific signal for a number of functions including switching from transcription to replication [31]. In HEV B viruses there is an addition Z domain that is well conserved among HEV B viruses and may be important for viral growth *in vivo*, but not *in vitro* [24, 32]. The resulting structure (Fig 9) was different than that proposed previously [24] for the HEV B consensus motif when nucleotide sequences specific for the iEV11 were mapped into this structure. Nucleotide differences found in one isolate, A13, would have further disrupted this structure in two positions and elongated a stem in another.

## Discussion

Normally enteroviruses cause acute infections that are cleared within few days or weeks. However, in rare cases, they can establish persistent infection lasting for years. Most known cases of chronic infection with enteroviruses involve patients with various kinds of primary immunodeficiency affecting B cell functions. However many aspects of this phenomenon remain poorly understood, including the cell and tissue localization, the factors controlling the outcome of virus-host interactions, and the reasons why the infection cannot be resolved. This current study was initiated in attempts to understand the evolution of the virus that persists in a patient chronically infected with EV11 who experienced periodic bouts of encephalitis that eventually led to death. This investigation was conducted within the context of patterns of nucleotide and amino acid differences observed among EV11 viruses isolated from sporadic and clustered EV11 infections in the community during the same time frame. The clinical course of this case differed from persistent poliovirus infections in immune deficient people who are largely asymptomatic. Our goal was to identify the source of infection and to shed some light on the environment the virus replicates in and the selective pressures it is experiencing. We have studied 10 iEV11 strains isolated over a period of almost 4 years from November 1995 till December 1999. To identify the source of the infection we have also sequenced strains of circulating EV11 isolated in Israel between 1992 and 1999. The material that was available to us included primary isolates and a cell culture passage performed in attempt to increase virus concentration. Virus in some samples lost its viability and could not be expanded in cell culture, and therefore the quantity of RNA that was available was insufficient for traditional Sanger sequencing of the entire genome. Therefore we have tried another approach based on deep sequencing using Illumina technology, which proved to be very effective and resulted in reconstruction of complete or near-complete consensus sequences and identification of sequence heterogeneities. This report demonstrates that deep sequencing methodology requires smaller amounts of starting material and is sequence-independent, i.e. does not require specific PCR primers used in traditional Sanger sequencing.

Comparison of the 25 genomic sequences generated in this study showed that four different genogroups circulated in Israel in the 1990s and that two of them co-circulated during the 1999 EV11 outbreak. Genogroup A viruses were isolated in 1992–1993, genogroup B in 1996–1997, genogroup C in 1998–1999, and genogroup D only in 1999. Co-circulation of two clusters of EV11 (C and D) in 1999 has been reported previously [5, 33].

All iEV11 strains isolated from the chronically infected patient clustered together with four cEV11 isolates of genogroup A. The virus that was the closest relative of the earliest (1995) iEV11 isolate was cEV11 strain isolated in 1992. They had 24 nucleotide differences in VP1-coding sequence (2.7%). Subsequent isolates gradually accumulated mutations so that the latest 1999 iEV11 isolate contained 51 differences (5.8%). The pace of this evolution (3.1% over a period of 3.8 years) is consistent with the rate of mutants accumulation established for circulating polioviruses [34]. The time plot of the number of nucleotide and amino acid differences in VP1 region was a straight line that could be extrapolated back to the early 1990s, consistent with the isolation date of the closest relative, and suggesting that the patient was infected at around that time with a contemporary cEV11 strain. Comparison of full-length sequences revealed no evidence of recombination with other viruses, and other parts of the viral genome also evolved linearly, albeit at a different pace.

Amino acid substitutions that occurred in cEV11 capsid differed from those that accumulated in iEV11. In circulating viruses mutations mostly occurred in the canyon and its southern rim (across from the 5-fold axis of symmetry), while in iEV11 many of them concentrated on the northern rim adjacent to the 5-fold axis. The canyon is the binding site for high affinity receptors that belong to the immunoglobulin superfamily, such as CD155, which is poliovirus receptor [35–37]. In contrast, EV11 and some other enteroviruses use Decay-Accelerating Factor (DAF, or CD55). DAF binds close to the 5-fold axis of symmetry on the surface and not in the canyon [38]. Interaction with DAF is low affinity with high on-off rates compared with canyon binding receptors capable of causing conformational changes of bound viruses [38–41]. For instance, EV11-DAF interaction has a dissociation constant KD = 3µM [40] whereas poliovirus-CD155 has KD = 80–700µM [42]. EV11 infection of polarized epithelial cells uses two different entry routes, directly from apical surface as well as tight junctions, which is dependent on DAF binding [39]. Preferential accumulation of mutations on the northern wall of the canyon and close to 5'-fold axis of symmetry during evolution of iEV11 suggests that the virus may be undergoing adaptation to DAF in specific cell types. This part of the virion surface is also known to harbor neutralizing epitopes, so the changes may also reflect immune evasion by the virus.

EV11 were the most sensitive of all enteroviruses to antiviral drug pleconaril [43]. Approximately two years after the onset of chronic infection the immunodeficient patient was treated with pleconaril in an attempt to clear the viral infection. Sequencing results revealed that the strain isolated from the patient at the time contained two mutations in VP1 (V_117_I and V_119_I) associated with EV11 resistance to pleconaril [44], explaining why the treatment attempt was unsuccessful.

Highly conserved RNA secondary structures rather than sequences are important for replication [32, 35]. Changes of nucleotides in essential structures such as domain V of the 5’UTR and the Z motif in the 3’UTR were observed for the iEV11s. Some nucleotide variations between isolates conserved the stem loop structures while others altered them.

The rate of accumulation of total nucleotide substitutions (Kt) in capsid regions of iEV11 and replicating poliovirus were similar [1] (0.76*10−2 and 1.03*10−2 mutations per site per year, respectively). However, the degree of amino acid sequences conservation was much lower in iEV11 than in poliovirus (Ka values of 0.72*10−2 and 0.03*10−2, respectively), suggesting that evolution of iEV11 was taking place under lower selective pressure. Respective values for cEV11 could not be calculated because of the scarcity of data and the unknown evolutionary trajectory of the few known sequences of cEV11. However, this conclusion was confirmed by comparing rates at which nucleotide and amino acid changes are fixed in different parts of viral genome during evolution. There are a total of 61 nucleotide triplets coding for 20 amino acids, therefore if the mutational process is random and there is no selective pressure restricting changes in the protein sequence, there should be 3 times more fixed nucleotide substitutions than fixed amino acid changes. Therefore comparison of densities of mutations in amino acid and nucleotide sequences (Da and Dn, the number of sites at which mutations are present divided by the length of protein and nucleotide sequences respectively) can reveal whether there is a positive or negative selective pressure. The Da/Dn ratio below 1 indicates that protein sequences are conserved, and that a disproportionately bigger number of mutations result in synonymous codons. A ratio greater than 1 may suggest that there is a positive selective pressure favoring accumulation of adaptive mutations. This analysis performed for iEV11 strains showed that in most viral proteins the ratio was close to 1, suggesting that they largely experience a random neutral evolution. The exception was viral 3C protease that was highly conserved and had only one mutation that changed isoleucine at amino acid 6 that is unique for genogroup A strains to methionine present in genogroups B, C, and D. In contrast, the pattern of selective pressure in cEV11 was different, and these viruses experienced higher selective pressure at amino acid level (the average Da/Dn ratio was 0.79 for iEV11 versus 0.22 for cEV11). Lower conservation of capsid proteins and different distribution of mutations on the capsid surface in iEV11 compared to cEV11 suggest that the virus may undergo adaptation to replication in different types of cells. Protease 3C that is highly conserved in iEV11 plays an important role in maturation of viral proteins as well as in cleaving several cellular proteins involved in innate antiviral defense [45]. Its conservation in iEV11 isolates may suggest that this function is important for viral persistence. Therefore establishment of chronic EV11 infection involves not only impairment of host immune system (B-cell immunodeficiency), bus also may require virus adaptation while preserving its functions mediating virus-host interactions.

## Supporting information

S1 Fig Frequency of EV11 isolation in Israel by year. (TIF)

S1 Table A list of EV11 isolates used for whole-genome deep sequencing and their origin. (DOCX)

S2 Table Amino acid differences in iEV11 relative to the closest Genogroup A isolate A01-EV11-5789 cEV-11. (DOCX)
