# Supplementary figures and images for: Evolution of echovirus 11 in a chronically infected immunodeficient patient
Source: PLoS Pathog. 2018 Mar 19;14(3):e1006943. doi: 10.1371/journal.ppat.1006943 (PMC5875893; doi:10.1371/journal.ppat.1006943)

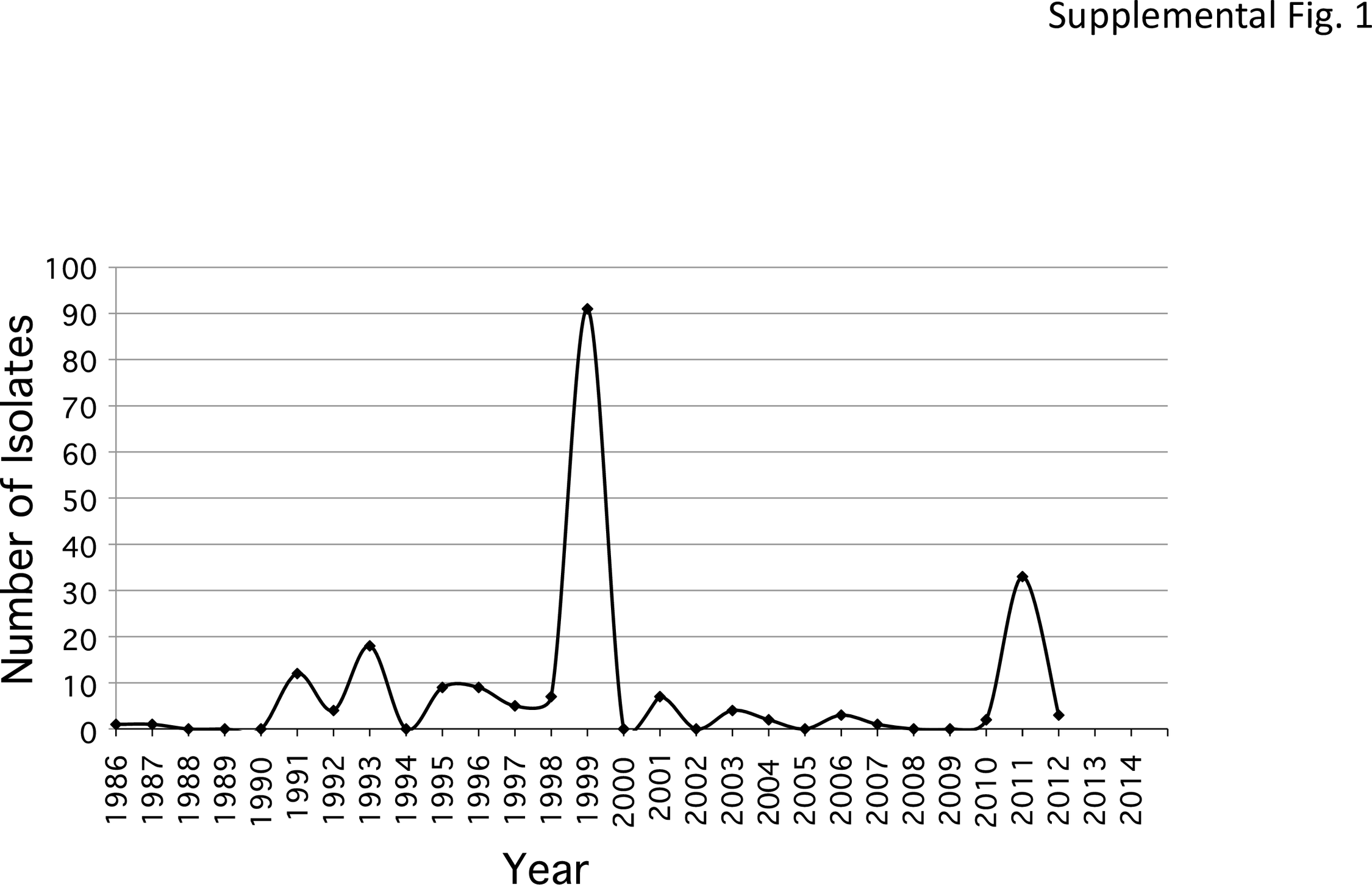

Supplement: S1 Fig — (TIF) [file ppat.1006943.s001.tif]
