# Supplementary material for: Evolution of echovirus 11 in a chronically infected immunodeficient patient
Source: PLoS Pathog. 2018 Mar 19;14(3):e1006943. doi: 10.1371/journal.ppat.1006943 (PMC5875893; doi:10.1371/journal.ppat.1006943)
Supplement: S1 Table — (DOCX) [file ppat.1006943.s002.docx]

| Supplemental Table 1. EV11 isolates used for whole-genome deep sequencing | | | | | | |  |  |  |  |
| --- | --- | --- | --- | --- | --- | --- | --- | --- | --- | --- |
| **Number** | **SampleID** | **Isolation date** | **Clinical sample** | **Source of virus** | **qPCR Ct** | **Notes** | **Clinical Reason for Referral or Outbreak Address** | **Sequence ID** | **Depth of sequencing coverage** | **Access Number** |
| 1 | 7482 | 13-Nov-95 | CSF | Original TC | 18.2 | Chronic excretor | Meningitis | A05_EV11-7482_ISR_13-Nov-1995 | 40 | KY981573 |
| 2 | 7676 | 27-Feb-96 | not listed | Passage | 15 | Chronic excretor | Not recorded | A06_EV11-7676_ISR_27-Feb-1996 | 4,815 | KY981574 |
| 3 | 8108 | 28-Oct-96 | CSF | Passage | 14.8 | Chronic excretor | Meningoencephalitis | A07_EV11-8108_ISR_28-Oct-1996 | 1,309 | KY981576 |
| 4 | 8416 | 06-May-97 | CSF | Original TC | 15.6 | Chronic excretor | Not recorded | A08_EV11-8416_ISR_06-May-1997 | 530 | KY981577 |
| 5 | 9295 | 11-Feb-98 | CSF | Passage | 14.3 | Chronic excretor | Meningoencephalitis | A10_EV11-9295_ISR_11-Feb-1998 | 1,526 | KY981579 |
| 6 | 9310 | 19-Feb-98 | CSF | Passage | 14.2 | Chronic excretor | Not recorded | A09_EV11-9310_ISR_19-Feb-1998 | 46,844 | KY981580 |
| 7 | 9368 | 01-Apr-98 | CSF | Passage | 14.1 | Chronic excretor | Not recorded | A11_EV11-9368_ISR_01-Apr-1998 | 59,244 | KY981581 |
| 8 | 1315 | 19-Oct-99 | CSF | Passage | 14.2 | Chronic excretor | Meningitis, encephalitis, paresis | A12_EV11-1315_ISR_19-Oct-1999 | 80,605 | KY981563 |
| 9 | 1373 | 01-Nov-99 | CSF | Original TC | 14.6 | Chronic excretor | Meningitis | A13_EV11-1373_ISR_01-Nov-1999 | 1,000 | KY981564 |
| 10 | 1541 | 21-Dec-99 | CSF | Passage | 14 | Chronic excretor | Meningoencephalitis, Temperature | A14_EV11-1541_ISR_21-Dec-1999 | 65,055 | KY981568 |
| 11 | 5789 | 1992 | rectal swab | Passage | 14.1 | Sporadic | EV compatible (diarrhea) | A01_EV11-5789_ISR_1992 | 7,845 | KY981569 |
| 12 | 5824 | 1992 | throat/nose | Passage | 14.1 | Sporadic | EV compatible (fever, cough, runny nose) | A02_EV11-5824_ISR_1992 | 7,938 | KY981570 |
| 13 | 5960 | 1993 | anal swab | Original TC | 17.1 | Sporadic | EV compatible (high fever) | A04_EV11-5960_ISR_1993 | 263,604 | KY981571 |
| 14 | 6067 | 1993 | CSF | Passage | 14.6 | Sporadic | EV compatible (neurological) | A03_EV11-6067_ISR_1993 | 18,630 | KY981572 |
| 15 | 8098 | 1996 | throat swab | Passage | 16.2 | Sporadic | Not recorded | B01_EV11-8098_ISR_1996 | 146,192 | KY981575 |
| 16 | 8640 | 1997 | stool | Passage | 16.3 | Sporadic | EV compatible (pericarditis) | B02_EV11-8640_ISR_1998 | 196,163 | KY981578 |
| 17 | 23 | 1998 | mucus | Original TC | 15.2 | Sporadic | EV compatible (URT infection) | C01_EV11-23_ISR_1997 | 2,369 | KY981557 |
| 18 | 1096 | 1999 | stool | Passage | 14.2 | Outbreak | Neve Chana | C04_EV11-1096_ISR_1999 | 287,121 | KY981562 |
| 19 | 1510 | 1999 | CSF | Passage | 14.1 | Outbreak | Rambam Med. Center | D03_EV11-1510_ISR_1999 | 274,018 | KY981565 |
| 20 | 674 | 1999 | CSF | Original TC | 15.3 | Outbreak | Mother and Child Clinic | C02_EV11-674_ISR_1999 | 65,025 | KY981559 |
| 21 | 535 | 1999 | stool | Original TC | 14.3 | Outbreak | Asaf Harofe Med. Center | D01_EV11-535_ISR_1999 | 84,622 | KY981558 |
| 22 | 1512 | 1999 | CSF | Passage | 14.6 | Outbreak | Rambam Med. Center | D04_EV11-1512_ISR_1999 | 212,507 | KY981566 |
| 23 | 1513 | 1999 | CSF | Passage | 14.3 | Outbreak | Rambam Med. Center | C05_EV11-1513_ISR_1999 | 125,661 | KY981567 |
| 24 | 675 | 1999 | CSF | Original TC | 15.4 | Outbreak | Mother and Child Clinic | C03_EV11-675_ISR_1999 | 131 | KY981560 |
| 25 | 1000 | 1999 | CSF | Original TC | 14.1 | Outbreak | Asaf Harofe Med. Center | D02_EV11-1000_ISR_1999 | 187 | KY981561 |
