# Supplementary material for: Evolution of echovirus 11 in a chronically infected immunodeficient patient
Source: PLoS Pathog. 2018 Mar 19;14(3):e1006943. doi: 10.1371/journal.ppat.1006943 (PMC5875893; doi:10.1371/journal.ppat.1006943)
Supplement: S2 Table — (DOCX) [file ppat.1006943.s003.docx]

| Supplemental Table 2. Amino acid differences in iEV11 relative to the closest Genogroup A isolate A01-EV11-5789 cEV-11 | | | | | | | | | | | | |  |
| --- | --- | --- | --- | --- | --- | --- | --- | --- | --- | --- | --- | --- | --- |
| Position in polyprotein | Mature peptide | Position in mature peptide | A01-cEV11-5789 | A05-EV11-7482 | A06 iEV11-7676 | A07 iEV11-8108 | A08-EV11-8416 | A09-EV11-9368 | A10-EV11-9295 | A11-EV11-9310 | A12-EV11-1541 | A13-EV11-1315 | A14-EV11-1373 |
| 18 | VP4 | 18 | N | S | S | S | S | S | S | S | S | S | S |
| 19 | VP4 | 19 | A | A | A | A | A | A | A | A | V | V | V |
| 24 | VP4 | 24 | I | I | I | I | I | I | I | I | T | T | T |
| 25 | VP4 | 25 | I | I | I | I | V | V | V | V | V | V | V |
| 62 | VP4 | 62 | I | I | I | I | V | V | V | V | V | V | V |
| 106 | VP2 | 37 | R | C | C | C | C | C | C | C | C | C | C |
| 143 | VP2 | 74 | D | G | G | G | G | G | G | G | G | G | G |
| 214 | VP2 | 145 | G | G | G | G | G | G | G | G | S | S | S |
| 217 | VP2 | 148 | E | D | D | D | D | D | D | D | D | D | D |
| 220 | VP2 | 151 | T | A | A | T | T | T | T | T | T | T | T |
| 223 | VP2 | 154 | A | V | V | V | V | V | V | V | V | V | V |
| 233 | VP2 | 164 | H | C | C | C | C | C | C | C | C | C | C |
| 297 | VP2 | 228 | S | S | P | P | P | P | P | P | P | P | P |
| 337 | VP3 | 6 | N | N | N | N | N | N | N | N | S | S | S |
| 390 | VP3 | 59 | E | E | E | K | E | E | E | E | E | E | E |
| 394 | VP3 | 63 | D | D | D | D | D | D | D | D | N | N | N |
| 428 | VP3 | 97 | H | R | R | R | R | H | R | R | H | H | H |
| 431 | VP3 | 100 | L | L | L | M | M | M | M | M | M | M | M |
| 445 | VP3 | 114 | V | V | V | V | V | I | I | I | I | I | I |
| 463 | VP3 | 132 | L | L | L | L | L | L | L | L | I | I | I |
| 478 | VP3 | 147 | D | G | G | G | G | G | G | G | G | G | G |
| 509 | VP3 | 178 | L | L | L | L | L | L | L | M | L | L | L |
| 520 | VP3 | 189 | N | N | N | N | Y | Y | Y | Y | Y | Y | Y |
| 545 | VP3 | 214 | V | V | V | V | V | A | A | A | A | A | A |
| 563 | VP3 | 232 | G | E | E | E | E | E | E | E | E | E | E |
| 573 | VP1 | 4 | V | V | M | M | M | M | M | M | M | M | M |
| 576 | VP1 | 7 | I | I | I | I | V | V | V | V | V | V | V |
| 616 | VP1 | 47 | T | I | I | I | I | I | I | I | I | I | I |
| 649 | VP1 | 80 | Y | C | C | C | C | C | C | C | C | C | C |
| 652 | VP1 | 83 | N | S | S | G | G | G | G | G | G | G | G |
| 685 | VP1 | 116 | D | D | N | N | N | N | N | N | D | D | D |
| 686 | VP1 | 117 | V | V | V | V | I | I | I | I | I | I | I |
| 688 | VP1 | 119 | V | V | V | V | I | I | I | I | I | I | I |
| 700 | VP1 | 131 | T | T | N | N | N | N | N | N | N | N | N |
| 701 | VP1 | 132 | Q | K | K | K | K | K | K | K | K | K | K |
| 713 | VP1 | 144 | I | L | L | L | L | L | L | L | L | L | L |
| 715 | VP1 | 146 | Y | F | F | F | F | F | F | F | F | F | F |
| 726 | VP1 | 157 | T | T | T | A | T | T | T | T | T | T | T |
| 809 | VP1 | 240 | F | F | F | F | F | F | F | F | F | F | L |
| 836 | VP1 | 267 | S | L | L | L | L | L | L | L | V | V | V |
| 843 | VP1 | 274 | K | G | G | G | G | G | G | G | G | G | G |
| 845 | VP1 | 276 | N | N | N | N | K | K | K | K | K | K | K |
| 850 | VP1 | 281 | I | I | I | I | V | V | V | V | V | V | V |
| 855 | VP1 | 286 | K | K | K | K | R | R | R | R | R | R | R |
| 859 | VP1 | 290 | S | L | L | L | S | S | S | S | S | S | S |
| 861 | VP1 | 292 | Y | H | H | H | R | R | R | R | R | R | R |
| 913 | 2A | 52 | I | V | V | V | V | V | V | V | V | V | V |
| 920 | 2A | 59 | M | T | T | T | T | T | T | T | T | T | T |
| 921 | 2A | 60 | A | A | A | A | A | T | T | T | T | T | T |
| 978 | 2A | 117 | E | K | K | K | K | K | K | K | K | K | K |
| 982 | 2A | 121 | I | V | V | V | V | V | V | V | V | V | V |
| 987 | 2A | 126 | M | L | L | L | L | L | L | L | L | L | L |
| 1078 | 2B | 67 | T | M | M | M | M | M | M | M | M | M | M |
| 1111 | 2C | 1 | S | S | S | S | S | G | G | G | G | G | G |
| 1112 | 2C | 2 | N | N | N | N | N | D | D | D | D | D | D |
| 1131 | 2C | 21 | I | I | V | I | I | I | I | I | I | I | I |
| 1228 | 2C | 118 | C | H | H | H | H | H | H | H | H | H | H |
| 1250 | 2C | 140 | S | N | N | N | N | S | S | S | S | S | S |
| 1346 | 2C | 236 | R | G | G | G | G | G | G | G | G | G | G |
| 1377 | 2C | 267 | K | R | G | A | A | A | A | A | A | A | A |
| 1406 | 2C | 296 | R | K | K | K | K | K | K | K | K | K | K |
| 1421 | 2C | 311 | F | L | L | L | L | L | L | L | L | L | L |
| 1443 | 3A | 4 | V | I | I | I | I | I | I | I | I | I | I |
| 1517 | 3A | 78 | I | V | V | V | V | V | V | V | V | V | V |
| 1534 | 3B | 6 | I | M | M | M | M | M | M | M | M | M | M |
| 1565 | 3C | 15 | A | T | T | T | A | A | A | A | A | A | A |
| 1747 | 3D | 14 | F | F | F | F | F | F | F | F | Y | Y | Y |
| 1752 | 3D | 19 | T | T | T | T | T | T | T | T | A | A | A |
| 1754 | 3D | 21 | S | S | S | S | S | C | C | C | C | C | C |
| 1756 | 3D | 23 | T | T | T | T | T | S | S | S | T | T | T |
| 1785 | 3D | 52 | A | A | A | A | T | A | A | A | A | A | A |
| 1786 | 3D | 53 | N | N | N | N | N | D | D | D | D | D | D |
| 1808 | 3D | 75 | Q | Q | Q | Q | Q | R | R | R | R | R | R |
| 1824 | 3D | 91 | N | N | N | N | N | N | N | N | S | S | S |
| 1869 | 3D | 136 | K | R | R | R | R | R | R | R | R | R | R |
| 1875 | 3D | 142 | K | K | K | K | K | R | R | R | R | R | R |
| 2033 | 3D | 300 | M | M | M | M | I | I | I | I | I | I | I |
| 2050 | 3D | 317 | I | I | I | I | I | I | I | I | V | V | V |
| 2083 | 3D | 350 | D | E | E | E | E | E | E | E | E | E | E |
| 2175 | 3D | 442 | I | V | V | V | V | V | V | V | V | V | V |
| 2180 | 3D | 447 | T | N | N | N | N | N | N | N | N | N | N |
|  |  |  |  |  |  |  |  |  |  |  |  |  |  |
|  |  |  |  |  |  |  |  |  |  |  |  |  |  |
|  |  |  |  |  |  |  |  |  |  |  |  |  |  |
|  |  |  |  |  |  |  |  |  |  |  |  |  |  |
|  |  |  |  |  |  |  |  |  |  |  |  |  |  |
|  |  |  |  |  |  |  |  |  |  |  |  |  |  |
|  |  |  |  |  |  |  |  |  |  |  |  |  |  |
|  |  |  |  |  |  |  |  |  |  |  |  |  |  |
|  |  |  |  |  |  |  |  |  |  |  |  |  |  |
|  |  |  |  |  |  |  |  |  |  |  |  |  |  |
|  |  |  |  |  |  |  |  |  |  |  |  |  |  |
|  |  |  |  |  |  |  |  |  |  |  |  |  |  |
|  |  |  |  |  |  |  |  |  |  |  |  |  |  |
